# Endoscopic flexure straightening: a novel technique for duodenal endoscopic submucosal dissection at difficult flexures

**DOI:** 10.1055/a-2971-3104

**Published:** 2026-10-09

**Authors:** Yuichi Hirano, Shunsuke Yoshii, Tomoki Michida, Ryu Ishihara

**Affiliations:** 1 Department of Gastrointestinal Oncology 53312 Osaka International Cancer InstituteOsakaOsaka PrefectureJapan

Endoscopic resection of duodenal tumors is increasingly common. However, lesions at the superior duodenal angle or inferior duodenal angle (IDA) remain technically challenging for duodenal endoscopic submucosal dissection (ESD), 1​ because the acute flexure limits access to the anal-side tumor margin. A laparoscopic-assisted technique to straighten such flexures has also been reported, 2​ but a purely endoscopic solution has been lacking. Here, we report two cases of duodenal ESD in which endoscopic flexure straightening (EFS) using an internal traction device enabled the successful completion of ESD ( Video 1 ). A schematic illustration of EFS is shown in Fig. 1 .

**Video 1** A novel technique for duodenal endoscopic submucosal dissection at difficult flexures: endoscopic flexure straightening.


**Fig. 1:**
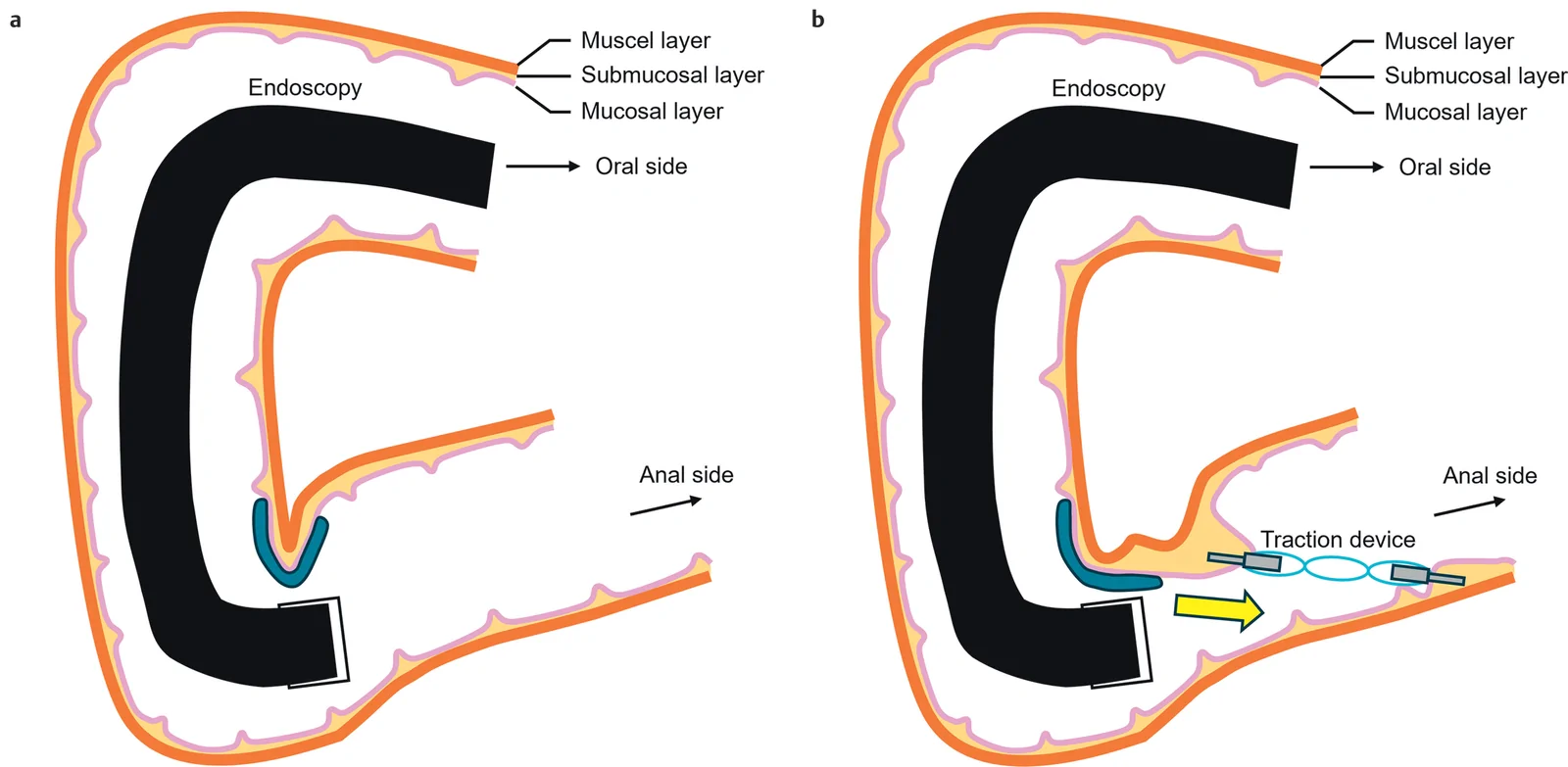
Schema of endoscopic flexure straightening. ( **a** ) The anal side of the lesion lies behind the flexure, limiting endoscopic access. ( **b** ) Straightening the flexural mucosa with a traction device improves endoscopic access.

Case 1 was a 59-year-old man with a 20-mm superficial non-ampullary duodenal epithelial tumor straddling the IDA. The acute flexure made the anal-side margin difficult to visualize ( Fig. 2a ). Before ESD, we attached a multi-loop traction device (MLTD; Boston Scientific, Tokyo, Japan) to the non-neoplastic mucosa on the anal side of the lesion and applied traction toward the contralateral mucosa ( Fig. 2b, c ). This maneuver straightened the flexure and allowed access to the anal margin of the lesion ( Fig. 2d ). After a circumferential incision, we dissected the submucosal layer and completed ESD. Pathology showed high-grade adenoma with negative lateral margins.

**Fig. 2:**
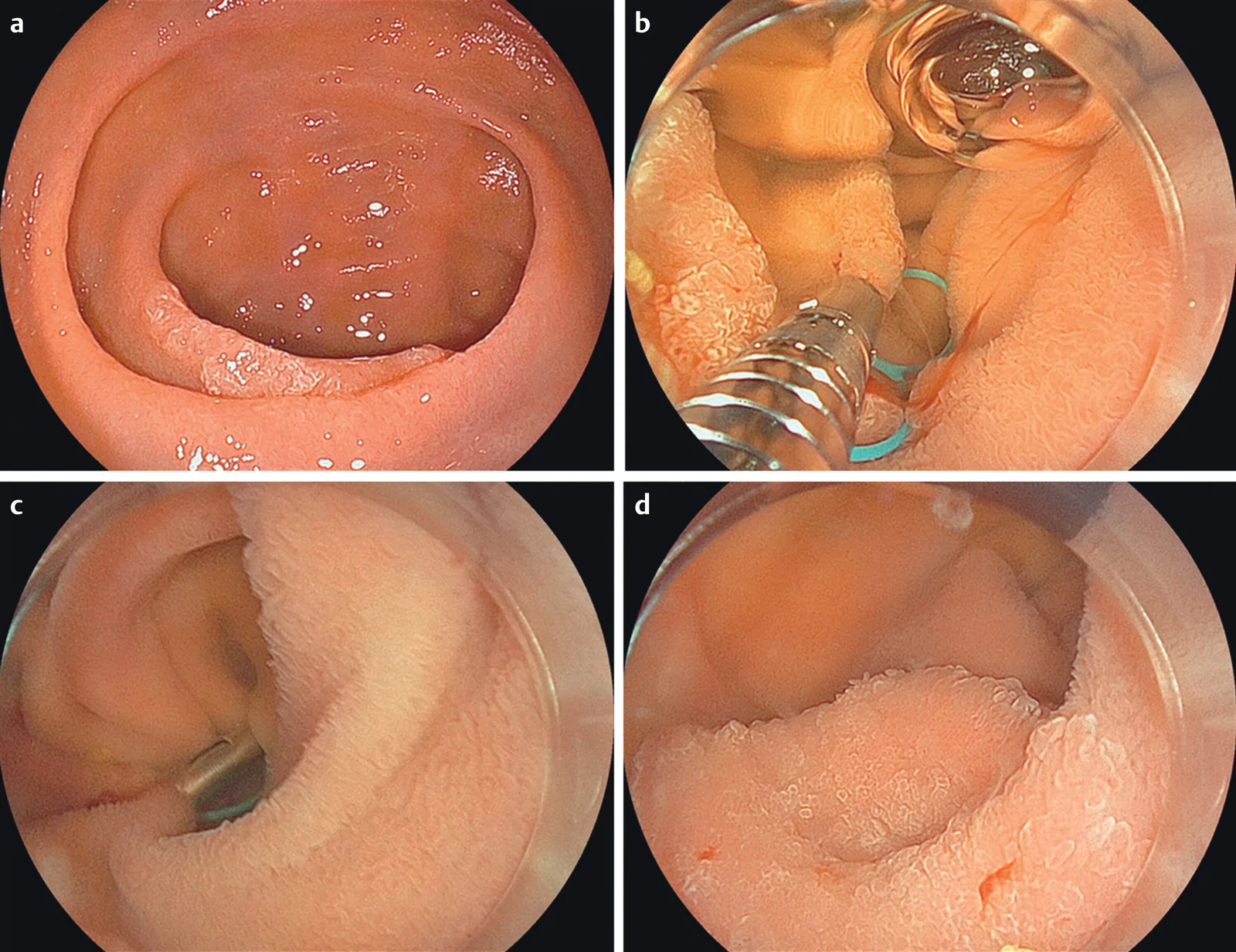
Endoscopic images of Case 1. ( **a** ) A 20-mm superficial non-ampullary duodenal epithelial tumor straddling the inferior duodenal angle (IDA). ( **b** ) A traction device was attached with a clip to non-neoplastic mucosa on the anal side of the lesion. ( **c** ) The traction device was further anchored with a second clip to the contralateral mucosa on the anal side. ( **d** ) The anal-side flexure was straightened, allowing the visualization of the lesion.

Case 2 was a 64-year-old man with a 70-mm ampullary tumor straddling the IDA. The acute flexure similarly limited access to the anal-side margin ( Fig. 3a ), so we performed EFS using an MLTD ( Fig. 3b ). This maneuver provided endoscopic access to the anal margin of the tumor ( Fig. 3c ), and we completed ESD including the papilla, without any adverse events. Pathology showed high-grade dysplasia with negative lateral margins ( Fig. 3d ).

**Fig. 3:**
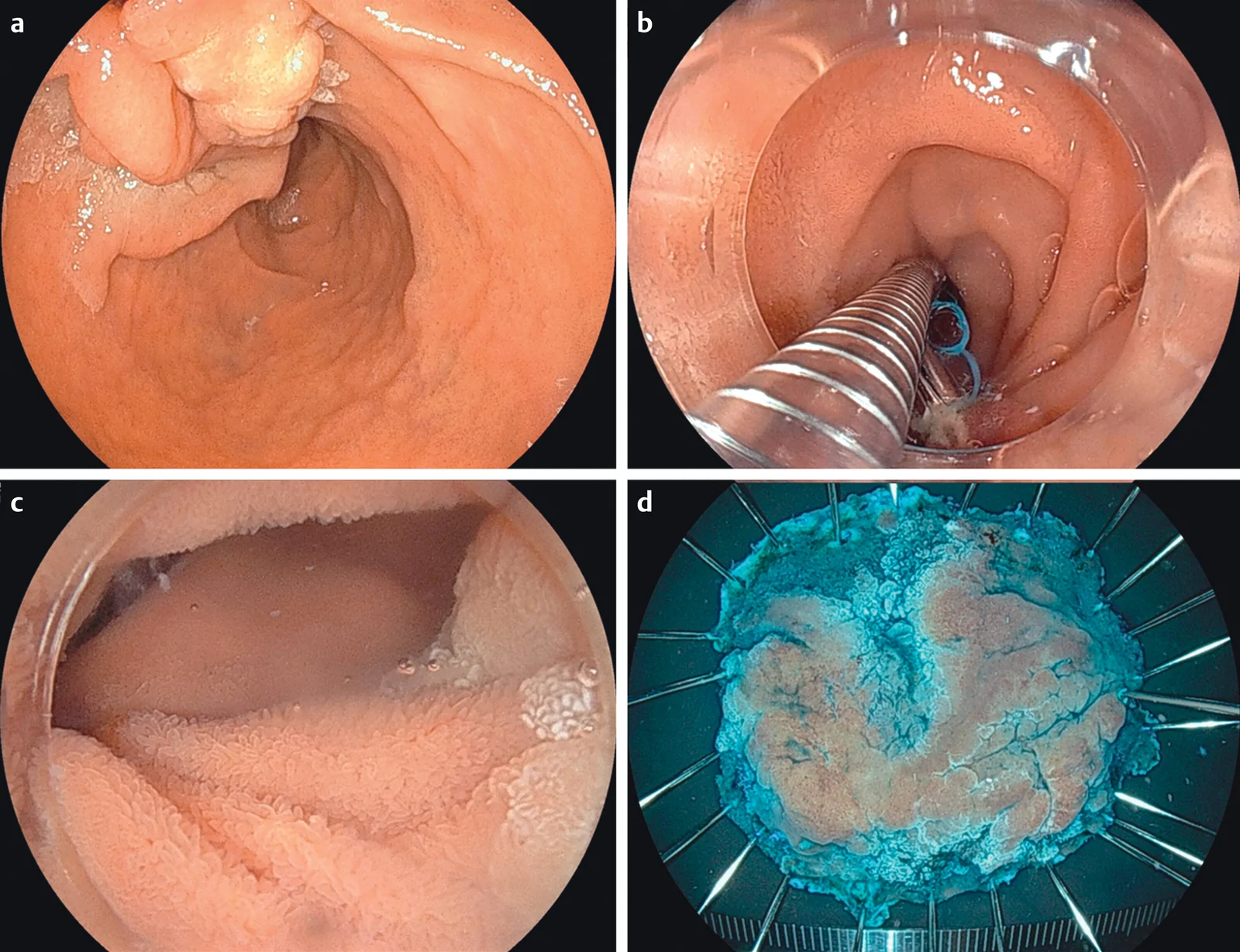
Endoscopic images of Case 2. ( **a** ) A 70-mm ampullary tumor straddling the IDA. ( **b** ) A traction device was attached with a clip to non-neoplastic mucosa on the anal side of the lesion and further anchored with a second clip to the contralateral mucosa on the anal side. ( **c** ) The anal-side flexure was straightened, allowing the visualization of the lesion. ( **d** ) En bloc resection with negative lateral margins was achieved.

EFS straightens acute duodenal flexures and improves access to otherwise difficult-to-reach tumor margins. This simple, purely endoscopic technique may expand the applicability of ESD to tumors located at difficult duodenal flexures.

Endoscopy_UCTN_Code_TTT_1AQ_2AD_3AD

## Data Availability

Data sharing is not applicable to this article.

## Declaration of GenAI Use

The authors confirm that no generative artificial Intelligence was used in the preparation of this manuscript.
